# Treatment patterns and clinical outcomes in patients with rheumatoid arthritis initiating etanercept, adalimumab, or Janus kinase inhibitor as first-line therapy: results from the real-world CorEvitas RA Registry

**DOI:** 10.1186/s13075-023-03120-9

**Published:** 2023-09-09

**Authors:** Dimitrios A. Pappas, Jacqueline O’Brien, Lin Guo, Ying Shan, Joshua F. Baker, Gregory Kricorian, Scott Stryker, David H. Collier

**Affiliations:** 1 CorEvitas LLC, Waltham, MA USA; 2 Corrona Research Foundation, Albany, NY USA; 3 Department of Rheumatology, Corporal Michael J. Crescenz Veterans Affairs Medical Center, Philadelphia, PA USA; 4 Amgen Inc., Thousand Oaks, CA USA

**Keywords:** Adalimumab, Biologic/targeted synthetic DMARDs, Etanercept, Janus kinase inhibitors, Patient-reported outcomes

## Abstract

**Background:**

Real-world studies assessing the comparative effectiveness of biologic or targeted synthetic disease-modifying antirheumatic drugs (b/tsDMARDs) as first-line targeted therapy are scarce. We analyzed the real-world persistence and effectiveness of etanercept (ETN), adalimumab (ADA), and Janus kinase inhibitors (JAKis) as first-line therapy in b/tsDMARD-naïve patients with rheumatoid arthritis (RA).

**Methods:**

Adults (≥ 18 years) enrolled in the CorEvitas RA Registry and initiating ETN, ADA, or a JAKi (alone or in combination with csDMARDs) between November 2012 and June 2021 were included if they had 6 and/or 12 months’ follow-up. Treatment persistence and effectiveness outcomes including the change in Clinical Disease Activity Index (CDAI) and patient-reported outcomes (PROs) were evaluated at follow-up, adjusting for covariates using linear and logistic regression models. An exploratory analysis for patients on monotherapy was also conducted.

**Results:**

Of 1059 ETN, 1327 ADA, and 581 JAKi initiators; 803 ETN, 984 ADA, and 361 JAKi initiators had 6 months’ follow-up. JAKi initiators were older and had a relatively longer disease duration than ETN or ADA initiators (mean age: 61.3 vs 54.5 and 55.5 years; mean duration of RA: 8.1 vs 5.7 and 5.6 years). Unadjusted mean improvements in CDAI and PROs were similar between the groups at 6 months, except the proportion achieving LDA, remission, and MCID in CDAI, which were numerically higher in the ETN and ADA groups vs JAKi group (LDA: 43.4% and 41.9% vs 32.5%; remission: 18.2% and 15.1% vs 11.5%; MCID: 46.5% and 47.8% vs 38.0%). Adjusted effectiveness results did not reveal statistically significant differences between treatment groups at 6 months, with an exception in MCID (odds ratio [95% CI] for JAKi vs ETN: 0.65 [0.43–0.98]). At 6 months, 68.2% of ETN, 68.5% of ADA, and 66.5% of JAKi initiators remained on therapy. The findings at 12 months’ follow-up and sensitivity analysis among monotherapy initiators also showed no differences in effectiveness outcomes between the groups.

**Conclusions:**

This analysis of real-world data from the CorEvitas RA Registry did not show differences in clinical effectiveness and treatment persistence rates in b/tsDMARD-naïve patients initiating ETN, ADA, or JAKi as first-line targeted therapy either alone or in combination with csDMARDs.

**Supplementary Information:**

The online version contains supplementary material available at 10.1186/s13075-023-03120-9.

## Background

The treatment landscape for rheumatoid arthritis (RA) has evolved substantially in the past two decades with the addition of biologic and targeted synthetic disease-modifying antirheumatic drugs (b/tsDMARDs) to the treatment armamentarium [1]. Currently available treatment options for the management of RA include conventional synthetic DMARDs (csDMARDs), bDMARDs (tumor necrosis factor inhibitors [TNFis] and non-TNFi biologics), and tsDMARDs (Janus kinase inhibitors [JAKis]) [1–3]. The 2021 American College of Rheumatology (ACR) guidelines for the management of RA strongly recommend initiating methotrexate (MTX) monotherapy over b/tsDMARD monotherapy for DMARD-naïve patients with moderate to high disease activity, and conditionally recommend the addition of a b/tsDMARD for patients who do not achieve remission or low disease activity (LDA) despite having maximally tolerated doses of MTX [2]. The 2022 European Alliance of Associations for Rheumatology (EULAR) guidelines also make similar recommendations with respect to the addition of a b/tsDMARD in patients who failed to achieve treatment target with csDMARDs [4]. The choice of the first b/tsDMARD after MTX failure is not mandated by the guidelines; in real-world, the choice is frequently based on factors such as patient and physician preferences and physician’s familiarity with the multiple b/tsDMARDs available [5, 6]. Randomized head-to-head trials are scarce, especially for tsDMARDs, which were approved for use in RA more recently [7–12]. In addition, studies on real-world comparative effectiveness are equally non-abundant and have reported conflicting findings [13–19]. Therefore, in this study, using data from the CorEvitas RA Registry, we compared the baseline characteristics, treatment persistence, and effectiveness of etanercept (ETN), adalimumab (ADA), and JAKis as first-line targeted therapy either alone or in combination with csDMARDs in b/tsDMARD-naïve patients with RA.

## Methods

### Data source and patient population

This was an observational study that used data from patients enrolled in the CorEvitas (formerly known as Corrona) RA Registry. The CorEvitas RA Registry is a longitudinal, multicenter, disease-based registry that collects data from patients and their treating rheumatologists during routine clinical encounters using standardized questionnaires [20]. Data on a wide variety of variables including demographics, socioeconomic and lifestyle characteristics, comorbidities, medication history with dates of use and reasons for therapy change, disease activity, patient-reported outcomes (PROs), adverse events, and other targeted safety outcomes are collected at each CorEvitas visit.

Biologic/tsDMARD-naïve patients aged ≥ 18 years with rheumatologist-confirmed diagnosis of RA and those who initiated treatment with ETN, ADA, or JAKi (tofacitinib, baricitinib, or upadacitinib) as first-line therapy between November 2012 and June 2021 with a 6- and/or 12-month follow-up visit were included. As the first JAKi (tofacitinib) was approved in November 2012, only those patients who initiated ETN and ADA during and after November 2012 were included in this analysis. The study was conducted following Good Pharmacoepidemiology Practices. All participating investigators obtained full institutional review board (IRB) approval for conducting non-interventional research involving human subjects. Sponsor approval and continuing review were obtained through a central IRB (New England Independent Review Board, NEIRB No. 120160610). For academic investigative sites that did not receive a waiver to use the central IRB, full board approval was obtained from the respective governing IRBs and documentation of approval was submitted to CorEvitas, LLC before initiating any study procedure. All participants provided written informed consent and authorization before participating.

### Outcomes and assessments

For this analysis, the index date was defined as the date of ETN, ADA, or JAKi initiation. The index visit was considered the registry visit during which the initiation took place. If the treatment initiation occurred in between CorEvitas registry visits, the visit preceding the initiation was considered as the index visit provided it was within 4 months from the actual drug initiation. Demographics and baseline clinical characteristics were ascertained at the index visit.

Outcomes were evaluated at 6 and 12 months after the index visit. A 6-month follow-up visit was a CorEvitas registry visit 3–9 months after the index date, and a 12-month follow-up visit was a CorEvitas visit 10–15 months after the index date. In cases where a patient had more than one visit that fell within a follow-up window, the visit closest to the 6- or 12-month date was chosen. Treatment effectiveness was assessed by the change from baseline (index or initiation visit) in disease activity and PRO scores. Clinical Disease Activity Index (CDAI), a composite index based on the summation of the tender joint count of 28 joints (TJC-28), swollen joint count of 28 joints (SJC-28), Physician’s Global Assessment of Disease Activity (PhGA), and Patient’s Global Assessment of Disease Activity (PtGA), was used for assessing disease activity [21]. The outcomes assessed at follow-up included the proportion of patients who remained on index therapy; change in disease activity and PROs from baseline; the proportion of patients achieving remission (CDAI ≤ 2.8) and LDA (CDAI ≤ 10) among those not in remission or with LDA at baseline, respectively; and the proportion achieving minimum clinically important difference (MCID) in CDAI, defined as a decrease in CDAI score of > 1, > 6, and > 12 for those in LDA (CDAI ≤ 10), moderate disease activity (MDA: CDAI > 10–22), and high disease activity (HDA: CDAI > 22) at baseline, respectively [22]. PROs evaluated included PtGA, patient pain, patient fatigue, morning stiffness hours, modified Health Assessment Questionnaire (mHAQ), and EuroQol-5D (EQ-5D).

### Statistical analysis

Categorical variables were presented as frequencies and percentages, and continuous variables were presented as means and standard deviations. The change in mean of outcomes was calculated by subtracting the value at the 6- or 12-month visit from the value at baseline (initiation visit). If an initiator discontinued therapy before the follow-up visit but did not switch therapy, the value at the 6- or 12-month follow-up visit was used. If an initiator switched to an alternative medication before the outcome evaluation visit, then the value at the time of the switch was used for the analysis if it was available for continuous outcomes. If the value of the continuous outcome at the time of switch was not available (e.g., in cases where switch occurred between the follow-up visits), the last value prior to switch was carried forward. For binary outcomes, if an initiator switched therapy before the follow-up visit, non-response was imputed.

Linear and logistic mixed-effects regression models were used to analyze the effectiveness outcomes adjusting for covariates that were imbalanced between treatment groups at baseline with absolute standardized mean difference of > 0.1 including demographics, socioeconomic and lifestyle characteristics, comorbidities, medication history, disease activity, and PROs. The investigation site was considered as a random factor to account for differences in prescribing patterns between sites in these models. Correlation coefficients with 95% confidence intervals (CIs), and odds ratios along with 95% CIs were provided for continuous and binary outcomes, respectively. An additional exploratory analysis of treatment persistence and effectiveness outcomes in a subset of patients receiving ETN, ADA, or JAKi as monotherapy during the study period was performed. Persistence to a drug was defined as the duration of time from initiation to discontinuation of therapy [23]. All analyses were performed using Stata Release 16 (StataCorp, College Station, TX).

## Results

### Demographics and clinical characteristics

**Fig. 1:**
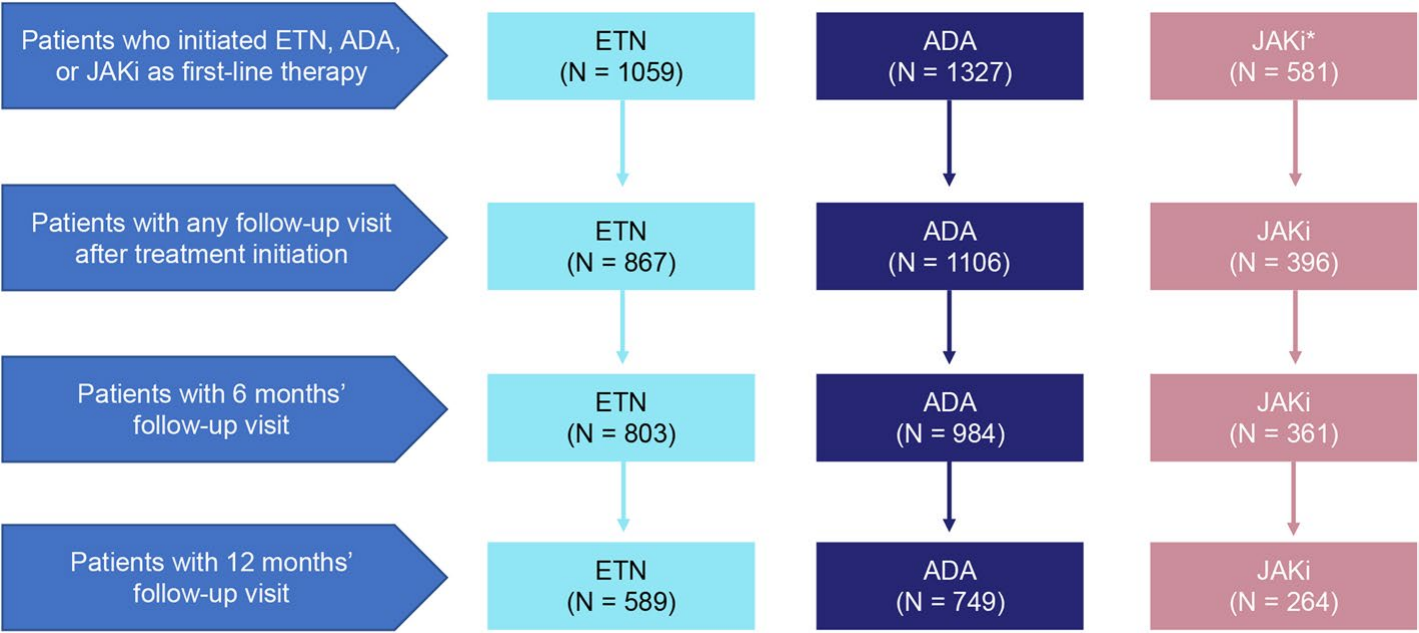
Patient attrition. *Over 80% of the patients in the JAKi group were on tofacitinib. ADA, adalimumab; ETN, etanercept; JAKi, Janus kinase inhibitor

Overall, 1059, 1327, and 581 b/tsDMARD-naïve patients initiated ETN, ADA, or a JAKi as first-line therapy during the study period, respectively. Of these initiators, the 6-month follow-up data were available for 803 (75.8%) ETN, 984 (74.2%) ADA, and 361 (62.1%) JAKi initiators, while the 12-month follow-up data were available for 589 (55.6%) ETN, 749 (56.4%) ADA, and 264 (45.4%) JAKi initiators (Fig. 1).

Among first-line initiators with 6 months of follow-up data, JAKi initiators were older and had a relatively longer disease duration compared with ETN or ADA initiators (mean age: 61.3 vs 54.5 and 55.5 years; mean disease duration: 8.1 vs 5.7 and 5.6 years) (Table 1). The mean number of csDMARDs received before initiating b/tsDMARD was > 1.5 in each group. A higher proportion of ETN and ADA initiators received these agents in combination with MTX and/or non-MTX non-biologic DMARDs than JAKi initiators (75.5% and 80.6% vs 67.6%). About half of the patients across the groups had a history of prednisone use (50.4%–53.7%), and the prevalence of comorbidities including cardiovascular disease and malignancies were higher in the JAKi initiator group than in the ETN and ADA groups. Disease activity and PRO scores were comparable between the groups at baseline (Table 1). The baseline characteristics of first-line initiators with 12 months of follow-up (Additional file 1: Table S1) were similar to those with 6 months of follow-up.

**Table 1:** Demographics and clinical characteristics at index visit for first-line ETN, ADA, and JAKi initiators with 6 months of follow-up

Characteristic	ETN initiators *N* = 803	ADA initiators *N* = 984	JAKi initiators *N* = 361
Age, years, mean (SD)	54.5 (13.1)	55.5 (12.1)	61.3 (12.4)
Female, n (%)	620 (77.2)	751 (76.3)	276 (76.5)
White, n/N (%)	640/797 (80.3)	811/976 (83.1)	300/357 (84.0)
BMI, kg/m^2^, mean (SD)	30.4 (7.6)	31.4 (7.9)	31.0 (7.4)
Duration of RA, years, mean (SD)	5.7 (7.5)	5.6 (7.4)	8.1 (9.7)
Rheumatoid factor positive, n/N (%)	344/503 (68.4)	420/640 (65.6)	130/216 (60.2)
CCP positive, n/N (%)	322/493 (65.3)	413/624 (66.2)	140/221 (63.3)
College education or above, n (%)	470 (60.8)	538 (56.8)	184 (53.3)
History of comorbidities, n (%)			
Cardiovascular disease	87 (10.8)	112 (11.4)	54 (15.0)
Malignancy	34 (4.2)	52 (5.3)	41 (11.4)
Serious infections	37 (4.6)	70 (7.1)	29 (8.0)
Fractures	198 (24.7)	308 (31.3)	108 (29.9)
Deep vein thrombosis/pulmonary embolism	12 (1.5)	17 (1.7)	8 (2.2)
Medication history			
Prior number of csDMARDs received (including current csDMARD), mean (SD)	1.5 (0.8)	1.8 (0.9)	1.7 (1.0)
History of prednisone use, n (%)	424 (52.8)	528 (53.7)	182 (50.4)
Current therapy	*n* = 803	*n* = 984	*n* = 361
Monotherapy, n (%)	197 (24.5)	191 (19.4)	117 (32.4)
Combination therapy, n (%)	606 (75.5)	793 (80.6)	244 (67.6)
MTX, n (%)	392 (48.8)	462 (47.0)	137 (38.0)
Non-MTX nbDMARDs,^a^ n (%)	127 (15.8)	180 (18.3)	71 (19.7)
MTX and non-MTX nbDMARDs,^a^ n (%)	87 (10.8)	151 (15.3)	36 (10.0)
Prednisone use, n (%)	217 (27.0)	270 (27.4)	89 (24.7)
Dose of prednisone, mg, mean (SD)	7.7 (5.4)	7.3 (6.2)	6.6 (3.7)
Disease activity and PROs, mean (SD)			
TJC-28	6.9 (7.1)	6.2 (6.5)	6.3 (6.8)
SJC-28	5.2 (5.5)	4.6 (5.0)	5.4 (5.3)
PhGA^b^	35.5 (24.2)	34.6 (22.9)	34.0 (22.9)
PtGA^b^	44.7 (27.2)	44.9 (26.6)	41.9 (26.8)
CDAI	20.0 (14.2)	18.8 (12.6)	19.3 (13.5)
Patient pain^b^	48.1 (28.7)	49.0 (28.3)	46.0 (29.6)
Patient fatigue^b^	47.4 (30.5)	47.6 (30.0)	45.3 (30.9)
mHAQ	0.5 (0.5)	0.5 (0.5)	0.5 (0.5)
EQ-5D	0.7 (0.2)	0.7 (0.2)	0.7 (0.2)
Patients with morning stiffness, n (%)	664 (87.8)	778 (86.3)	278 (83.0)
Duration of morning stiffness, hours, mean (SD)	2.1 (3.8)	2.0 (3.2)	2.1 (4.0)

“n” represents the number of patients with available data at the index visit

^a^nbDMARDs include methotrexate, hydroxychloroquine, leflunomide, sulfasalazine, azathioprine, minocycline, and cyclosporine

^b^Visual analog scale (0–100)

*ADA *Adalimumab, *BMI *Body mass index, *CCP *Cyclic citrullinated peptide, *CDAI *Clinical Disease Activity Index, *csDMARD *Conventional synthetic disease-modifying antirheumatic drug, *EQ-5D *EuroQol-5D, *ETN *Etanercept, *JAKi *Janus kinase inhibitor, *mHAQ *Modified Health Assessment Questionnaire, *MTX *Methotrexate, *nbDMARD* non-biologic disease-modifying antirheumatic drug, *PhGA *Physician’s Global Assessment of Disease Activity, *PRO *Patient-reported outcome, *PtGA *Patient’s Global Assessment of Disease Activity, *RA *Rheumatoid arthritis, *SD *Standard deviation, *SJC-28 *Swollen joint count of 28 joints, *TJC-28 *Tender joint count of 28 joints

An exploratory analysis was conducted on a subset of patients receiving these treatments as monotherapy (*n* = 550; ETN: 218, ADA: 212, and JAKi: 120 [> 85% were on tofacitinib]). Of these patients, 197 (90.4%) ETN, 191 (90.1%) ADA, and 117 (97.5%) JAKi initiators had a follow-up visit at 6 months, while 136 (62.4%) ETN, 143 (67.5%) ADA, and 77 (64.2%) JAKi initiators had a follow-up visit at 12 months. The majority of the demographic and disease characteristics in this subgroup were similar to the overall population with some exceptions as shown in Table 2. The baseline characteristics in the cohort of first-line monotherapy initiators with 12 months of follow-up (Additional file 1: Table S2) were similar to monotherapy initiators with 6 months of follow-up.

**Table 2:** Demographics and clinical characteristics at index visit for first-line ETN, ADA, and JAKi monotherapy initiators with 6 months of follow-up

Characteristic	ETN initiators *N* = 197	ADA initiators *N* = 191	JAKi initiators *N* = 117
Age, years, mean (SD)	52.4 (13.2)	54.3 (11.1)	62.5 (12.0)
Women, n (%)	155 (78.7)	140 (73.3)	90 (76.9)
White, n/N (%)	160/196 (81.6)	156/189 (82.5)	102/117 (87.2)
BMI, kg/m^2^, mean (SD)	30.2 (7.7)	31.1 (7.1)	30.5 (6.6)
Duration of RA, years, mean (SD)	5.7 (7.3)	5.3 (6.3)	7.3 (8.9)
Rheumatoid factor positive, n/N (%)	83/118 (70.3)	72/118 (61.0)	34/64 (53.1)
CCP positive, n/N (%)	80/119 (67.2)	73/117 (62.4)	42/70 (60.0)
College education or above, n (%)	115 (60.2)	100 (55.6)	56 (49.6)
History of comorbidities, n (%)			
Cardiovascular disease	16 (8.1)	25 (13.1)	12 (10.3)
Malignancy	4 (2.0)	11 (5.8)	13 (11.1)
Serious infections	6 (3.0)	15 (7.9)	5 (4.3)
Fractures	49 (24.9)	57 (29.8)	36 (30.8)
Deep vein thrombosis/pulmonary embolism	1 (0.5)	2 (1.0)	2 (1.7)
Medication history			
Prior number of csDMARDs received (including current csDMARD), mean (SD)	1.2 (0.9)	1.6 (1.1)	1.4 (1.0)
History of prednisone use, n (%)	94 (47.7)	99 (51.8)	54 (46.2)
Prednisone use, n (%)	51 (25.9)	48 (25.1)	27 (23.1)
Dose of prednisone, mg, mean (SD)	8.3 (5.2)	8.9 (9.0)	7.9 (5.1)
Disease activity and PROs, mean (SD)			
TJC-28	6.1 (6.2)	5.5 (5.8)	7.2 (8.1)
SJC-28	4.6 (5.1)	3.5 (4.4)	5.5 (5.6)
PhGA^a^	37.1 (23.8)	31.3 (23.5)	31.6 (23.7)
PtGA^a^	46.6 (26.2)	46.7 (26.5)	41.9 (28.9)
CDAI	19.2 (12.7)	16.8 (11.6)	20.0 (15.7)
Patient pain^a^	49.4 (28.4)	52.5 (29.7)	42.8 (30.7)
Patient fatigue^a^	45.7 (31.1)	51.4 (30.3)	44.2 (31.4)
mHAQ	0.5 (0.5)	0.6 (0.5)	0.5 (0.5)
EQ-5D	0.7 (0.2)	0.7 (0.2)	0.7 (0.2)
Patients with morning stiffness, n (%)	162 (89.5)	150 (85.2)	89 (84.8)
Duration of morning stiffness, hours, mean (SD)	2.4 (4.2)	2.2 (3.7)	2.2 (4.3)

“n” represents the number of patients with available data at the index visit

^a^Visual analog scale (0–100)

*ADA* Adalimumab, *BMI* Body mass index, *CCP* Cyclic citrullinated peptide, *CDAI* Clinical Disease Activity Index, *csDMARD* Conventional synthetic disease-modifying antirheumatic drug, *EQ-5D* EuroQol-5D, *ETN* Etanercept, *JAKi* Janus kinase inhibitor, *mHAQ* Modified Health Assessment Questionnaire, *PhGA* Physician’s Global Assessment of Disease Activity, *PRO* patient-reported outcome, *PtGA* Patient’s Global Assessment of Disease Activity, *RA* Rheumatoid arthritis, *SD* Standard deviation, *SJC-28* Swollen joint count of 28 joints, *TJC-28* Tender joint count of 28 joints

### Treatment persistence

**Fig. 2:**
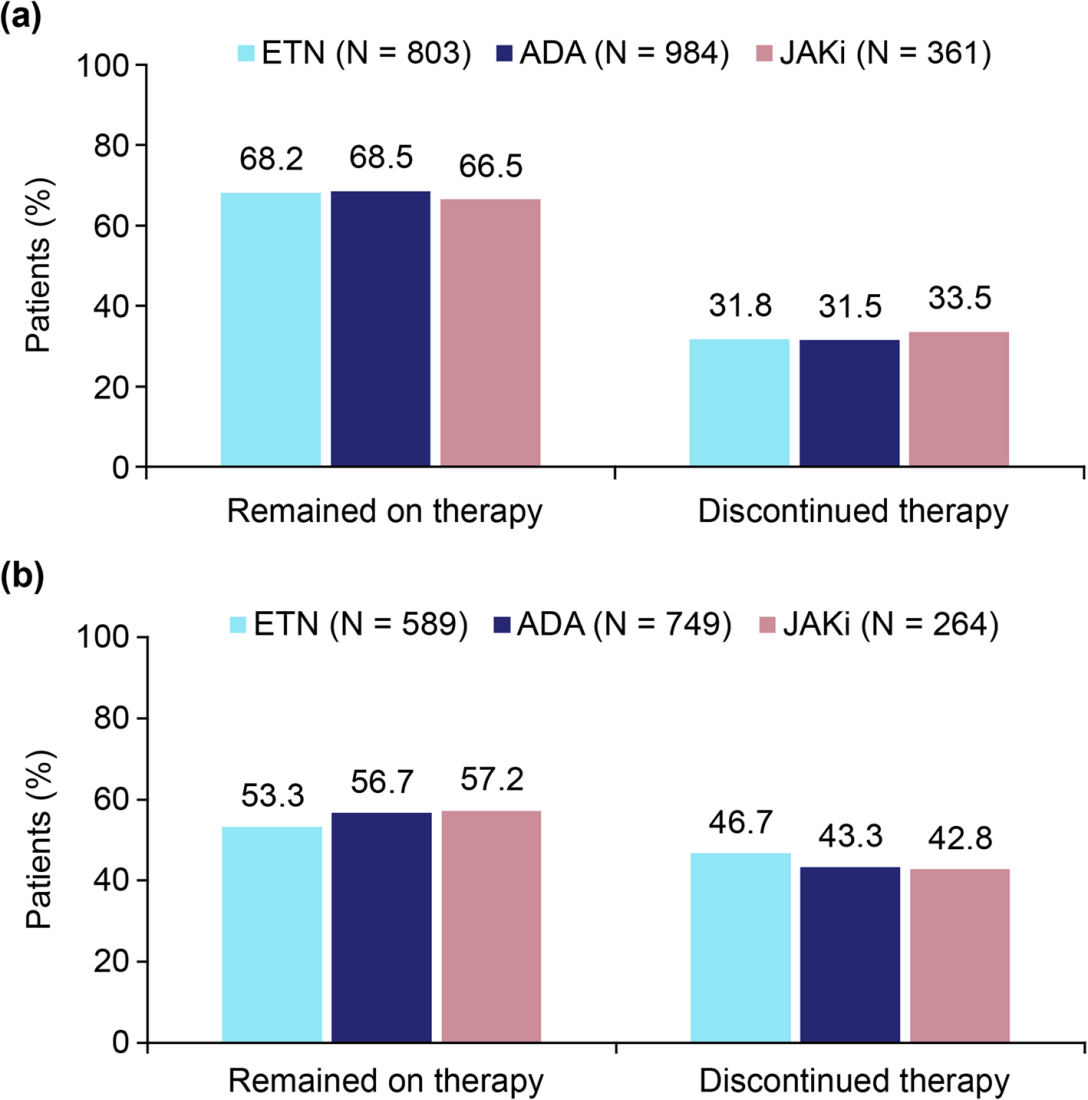
Treatment persistence at 6-month (**a**) and 12-month (**b**) follow-up among first-line initiators of ETN, ADA, and JAKis. Patients who discontinued therapy included those who had withdrawn therapy but not switched and those who switched to another b/tsDMARD. ADA, adalimumab; b/tsDMARD, biologic/targeted synthetic disease-modifying antirheumatic drug; ETN, etanercept; JAKi, Janus kinase inhibitor

Results from unadjusted analysis showed that around two-thirds of patients receiving ETN, ADA, and JAKis remained on these therapies at 6 months with no major differences observed between treatment groups (Fig. 2). Nearly one-third of the patients across groups (31.5%–33.5%) discontinued therapy at the 6-month follow-up. Among patients with available data on the reasons for discontinuation of treatments, efficacy-related reasons were less frequent in the JAKi initiator group compared with those on ETN and ADA, while no notable differences were observed between groups in the proportion of patients who discontinued therapy due to safety reasons at the 6-month follow-up (Additional file 1: Table S3). At 12 months, over half of the patients remained on therapy across groups (53.3%–57.2%).

**Fig. 3:**
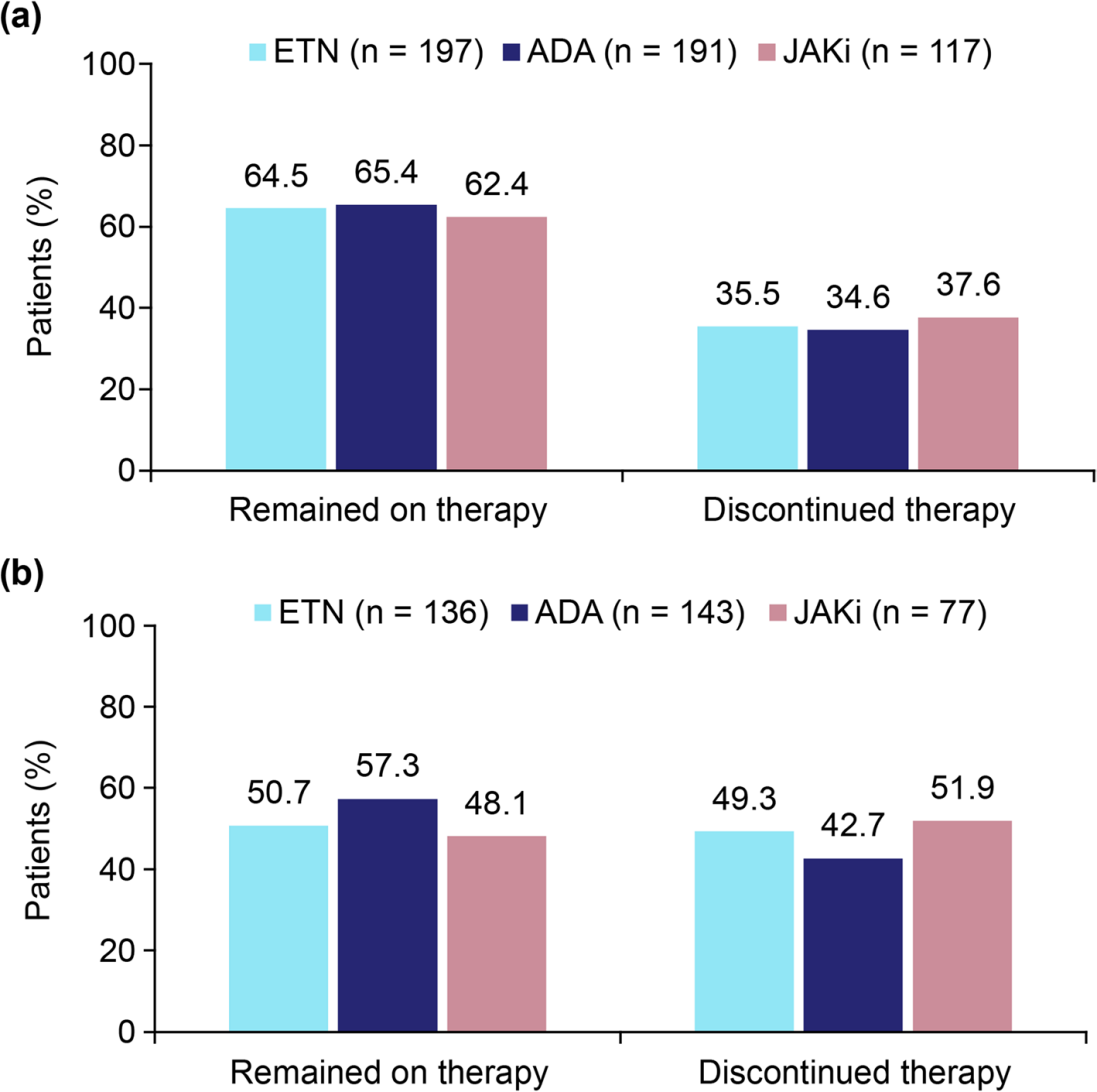
Treatment persistence at 6-month (**a**) and 12-month (**b**) follow-up among first-line monotherapy initiators of ETN, ADA, and JAKis. Patients who discontinued therapy included those who had withdrawn therapy but not switched and those who switched to another b/tsDMARD. ADA, adalimumab; b/tsDMARD, biologic/targeted synthetic disease-modifying antirheumatic drug; ETN, etanercept; JAKi, Janus kinase inhibitor

Among monotherapy initiators, the proportion who remained on therapy at 6 months was similar to that observed in the overall population at the 6-month follow-up (Fig. 3). However, at 12 months, a slightly higher proportion of ADA initiators remained on therapy (57.3% vs 50.7% and 48.1% for ETN and JAKi initiators, respectively; Fig. 3).

### Disease activity and PROs

The mean improvements in disease activity and PROs were similar between treatment groups at 6 and 12 months in both adjusted and unadjusted analysis (Table 3 and Additional file 1: Table S4). Results from the unadjusted analysis showed that at 6 months, 43.4% in ETN, 41.9% in ADA, and 32.5% in JAKi group achieved LDA; 18.2% in ETN, 15.1% in ADA, and 11.5% in JAKi group achieved remission; 46.5% in ETN, 47.8% in ADA, and 38.0% in JAKi group achieved clinically meaningful improvement in CDAI, while no major differences were observed in the proportion achieving these outcomes at 12 months. After adjusting for baseline covariates, the odds of achieving remission and LDA did not differ between treatment groups at 6 and 12 months. At 6 months, JAKi initiators were less likely to achieve MCID in CDAI (OR [95% CI] vs ETN: 0.65 [0.43–0.98]) in the adjusted analysis, while at 12 months the likelihood of achieving MCID in CDAI was similar to that in other treatment groups (Table 3). PROs including patient pain, patient fatigue, mHAQ, EQ-5D, and morning stiffness showed no major difference between groups at both time points in the adjusted analysis.

**Table 3:** Adjusted change in disease activity and PROs among first-line ADA and JAKi initiators at 6 and 12 months of follow-up relative to ETN

Outcome	At 6 months^a^		At 12 months^b^	
	**ADA initiators**	**JAKi initiators**	**ADA initiators**	**JAKi initiators**
CDAI	–0.08 (–1.48, 1.33)	–0.65 (–2.45, 1.15)	0.05 (–1.59, 1.69)	0.22 (–1.83, 2.27)
TJC-28	–0.08 (–0.82, 0.66)	–0.49 (–1.43, 0.46)	–0.17 (–1.06, 0.73)	–0.16 (–1.28, 0.96)
SJC-28	0.09 (–0.50, 0.69)	0.09 (–0.67, 0.86)	0.20 (–0.48, 0.88)	–0.21 (–1.06, 0.65)
PhGA	0.10 (–2.79, 2.99)	–1.04 (–4.74, 2.66)	–0.36 (–3.95, 3.24)	0.59 (–3.90, 5.08)
PtGA	0.01 (–3.69, 3.71)	–2.99 (–7.75, 1.77)	1.41 (–3.07, 5.89)	0.02 (–5.58, 5.62)
mHAQ	–0.01 (–0.07, 0.04)	–0.07 (–0.14, 0.00)	–0.03 (–0.10, 0.03)	–0.08 (–0.17, 0.00)
Patient pain	0.09 (–3.88, 4.06)	–1.10 (–6.21, 4.00)	2.71 (–2.16, 7.58)	0.33 (–5.79, 6.44)
Patient fatigue	–3.09 (–6.96, 0.78)	1.44 (–3.52, 6.39)	–0.13 (–5.00, 4.75)	4.26 (–1.82, 10.34)
EQ-5D	0.00 (–0.02, 0.02)	–0.01 (–0.04, 0.02)	–0.02 (–0.05, 0.01)	–0.01 (–0.05, 0.02)
Morning stiffness hours	–0.26 (–0.77, 0.25)	–0.26 (–0.92, 0.40)	0.21 (–0.40, 0.83)	–0.10 (–0.88, 0.68)
Achievement of LDA,^c^ OR (95% CI)	1.09 (0.73, 1.62)	0.74 (0.44, 1.24)	0.90 (0.55, 1.47)	0.83 (0.45, 1.52)
Achievement of remission,^d^ OR (95% CI)	1.13 (0.73, 1.74)	0.77 (0.41, 1.43)	0.96 (0.57, 1.63)	0.88 (0.44, 1.75)
Achievement of MCID in CDAI,^e^ OR (95% CI)	1.10 (0.81, 1.50)	0.65 (0.43, 0.98)	1.01 (0.71, 1.45)	1.25 (0.79, 1.97)

Values represent regression coefficients and 95% CIs, unless otherwise specified. A positive value for adjusted change indicates a larger degree of improvement relative to the ETN reference

^a^Adjusted by baseline covariates including age, gender, rheumatoid factor positive status, college education, work status, private insurance, Medicare status, weight, history of comorbidities (CVD, hypertension, malignancy, serious infections, osteoporosis, and fractures), history of csDMARDs used, current therapy monotherapy or combination therapy, and CDAI

^b^Adjusted by baseline covariates including age, gender, cyclic citrullinated peptide positive status, race (Black), college education, work status, private insurance, Medicare status, smoking status, weight, history of comorbidities (hypertension, malignancy, serious infections, osteoporosis, and fractures), history of csDMARDs used, current prednisone use, current therapy (monotherapy or combination therapy), and CDAI

^c^CDAI score ≤ 10 among those with moderate or high disease activity at baseline

^d^CDAI score ≤ 2.8 among those with low disease activity or more severe disease activity

^e^MCID was defined as a decrease in CDAI score of > 1, > 6, and > 12 for those in LDA (CDAI ≤ 10), MDA (CDAI > 10–22), and HDA (CDAI > 22) at baseline, respectively

*ADA* Adalimumab, *CDAI* Clinical Disease Activity Index, *CI* Confidence interval, *csDMARD* Conventional synthetic disease-modifying antirheumatic drug, *CVD* Cardiovascular disease, *EQ-5D* EuroQol-5D, *ETN* Etanercept, *JAKi* Janus kinase inhibitor, *LDA* Low disease activity, *MCID* Minimum clinically important difference, *mHAQ* Modified Health Assessment Questionnaire, *OR* Odds ratio, *PhGA* Physician’s Global Assessment of Disease Activity, *PRO* Patient-reported outcome, *PtGA* Patient’s Global Assessment of Disease Activity, *SJC-28* Swollen joint count of 28 joints, *TJC-28* Tender joint count of 28 joints

Among monotherapy initiators, similar to the findings in the overall population, more patients achieved LDA with ETN at 6 and 12 months in the unadjusted analysis (Additional file 1: Table S5). After adjusting for covariates, no differences were observed in the disease activity and PROs including patient pain, patient fatigue, mHAQ, EQ-5D, and morning joint stiffness at the 6- and 12-month follow-up. The odds of achieving remission, LDA, and MCID in CDAI were also similar between groups at both time points (Additional file 1: Table S6).

## Discussion

Using data from a large national prospective registry, this real-world observational study analyzed treatment persistence and effectiveness of ETN, ADA, or a JAKi as first-line b/tsDMARD therapy and found that the treatment persistence rates and improvements in disease activity and PROs were similar between first-line ETN, ADA, and JAKi initiators.

Previous studies that compared the efficacy of JAKi vs ADA have shown disparity in findings: studies including the ORAL Standard [11, 24], ORAL Strategy [8, 25], and FINCH1 [7] reported similar efficacy between JAKi and ADA, and studies such as RA-BEAM [10] and SELECT-COMPARE [9, 26] reported superiority of JAKi over ADA in improving RA symptoms and PROs.

In the current study, we assessed treatment effectiveness by evaluating the change in CDAI score from baseline and the proportion achieving CDAI-based LDA and remission, and found no differences between ETN, ADA, and JAKi groups at 6- and 12-months follow-up in an adjusted analysis after adjusting for age, gender, history of comorbidities, CDAI, and other covariates. Moreover, no differences were observed between groups for PROs including patient pain, patient fatigue, mHAQ, EQ-5D, and morning stiffness. These findings are consistent with those of prior analysis of the CorEvitas registry [18], which showed no statistically significant differences between TNFi, non-TNFi, or tsDMARD groups for outcomes including changes in CDAI score, achievement of remission/LDA by CDAI and modified Disease Activity Score of 28 joints (DAS28), achievement of MCID in CDAI, HAQ-Disability Index, and other PROs in b/tsDMARD-naïve patients. In a retrospective analysis using administrative claims data from the IBM MarketScan Commercial Claims and Encounters Database, Gharaibeh et al., assessed the 12-month effectiveness of TNFis, non-TNFi biologics, and tofacitinib as first-line therapy using a validated claims-based effectiveness algorithm [17]. The study found that the proportion of patients effectively treated with ETN (31.4%) was similar to that treated with ADA (30.9%) and was relatively higher than that treated with tofacitinib (26.0%), with non-adherence being the main reason for the failing effectiveness of the therapies. In the current study, we found similar treatment persistence rates for ETN, ADA, and JAKis at the 6- and 12-month follow-up when used as first-line therapy either alone or in combination with csDMARDs in biologic-naïve patients. The findings from our study and the above-mentioned studies demonstrate the similar effectiveness of ETN, ADA, and tofacitinib when used as first-line targeted therapy in b/tsDMARD-naïve patients with RA.

As with all registry-based studies, our study has some strengths and limitations. To the best of our knowledge, this is the largest study comparing treatment persistence and effectiveness of the most commonly used TNFis (ETN and ADA) and JAKis administered either alone or in combination with csDMARDs in b/tsDMARD-naïve patients. In addition, the patient population in the CorEvitas RA registry has been shown to be representative of the general US population [27]. The limitations of this study include the lower proportion of patients with follow-up in the JAKi group than in other groups, possibly because the follow-up visits may have fallen out of the accepted follow-up windows, or not enough time had elapsed to be eligible for follow-up for those initiating the newly approved JAKis; the risk of residual confounding, such as confounding by indication, with JAKis being prescribed more commonly as a later line of therapy; and the lack of generalizability of the findings from this US-based registry to regions outside of the US.

## Conclusions

No differences in effectiveness and treatment persistence rates were observed in b/tsDMARD-naïve patients who initiated ETN, ADA, or a JAKi as first-line targeted therapy either alone or in combination with csDMARDs. These real-world observations suggest that multiple approaches to initial biologic therapy are appropriate for b/tsDMARD-naïve patients with RA.

### Supplementary Information

**Additional file 1: Table S1.** Demographics and clinical characteristics at index visit for first-line ETN, ADA, and JAKi initiators with 12 months of follow-up. **Table S2.** Demographics and clinical characteristics at index visit for first-line ETN, ADA, and JAKi monotherapy initiators with 12 months of follow-up. **Table S3**. Reasons for discontinuation of treatment among ETN, ADA, and JAKi initiators at 6- and 12-months follow-up. **Table S4.** Unadjusted change in disease activity and PROs among first-line initiators of ETN, ADA, or JAKi at 6- and 12- months of follow-up. **Table S5.** Unadjusted change in disease activity and PROs among first-line initiators of ETN, ADA, and JAKi monotherapy at 6- and 12-months of follow-up. **Table S6.** Adjusted change in disease activity and PROs among first-line initiators of ADA and JAKi monotherapy at 6- and 12- months of follow-up relative to ETN.

## Data Availability

Data are available from CorEvitas, LLC through a commercial subscription agreement and are not publicly available. No additional data are available from the authors. Qualified researchers may request data from Amgen clinical studies. Complete details are available at the following: https://www.amgen.com/science/clinical-trials/clinical-data-transparency-practices/clinical-trial-data-sharing-request

## Abbreviations

ACR: American College of Rheumatology
ADA: Adalimumab
b/tsDMARD: Biologic or targeted synthetic disease-modifying antirheumatic drug
BMI: Body mass index
CCP: Cyclic citrullinated peptide
CDAI: Clinical Disease Activity Index
CI: Confidence interval
csDMARD: Conventional synthetic disease-modifying antirheumatic drug
CVD: Cardiovascular disease
EQ-5D: EuroQol-5D
ETN: Etanercept
EULAR: European Alliance of Associations for Rheumatology
HDA: High disease activity
IRB: Institutional review board
JAKi: Janus kinase inhibitor
LDA: Low disease activity
MCID: Minimum clinically important difference
MDA: Moderate disease activity
mHAQ: Modified Health Assessment Questionnaire
MTX: Methotrexate
PhGA: Physician’s Global Assessment of Disease Activity
PRO: Patient-reported outcome
PtGA: Patient’s Global Assessment of Disease Activity
RA: Rheumatoid arthritis
SD: Standard deviation
SJC-28: Swollen joint count of 28 joints
TJC-28: Tender joint count of 28 joints
TNFi: Tumor necrosis factor inhibitor
