# Supplementary material for: Treatment patterns and clinical outcomes in patients with rheumatoid arthritis initiating etanercept, adalimumab, or Janus kinase inhibitor as first-line therapy: results from the real-world CorEvitas RA Registry
Source: Arthritis Res Ther. 2023 Sep 9;25:166. doi: 10.1186/s13075-023-03120-9 (PMC10492389; doi:10.1186/s13075-023-03120-9)
Supplement: Supplementary file 1 — Additional file 1: Table S1. Demographics and clinical characteristics at index visit for first-line ETN, ADA, and JAKi initiators with 12 months of follow-up. Table S2. Demographics and clinical characteristics at index visit for first-line ETN, ADA, and JAKi monotherapy initiators with 12 months of follow-up. Table S3. Reasons for discontinuation of treatment among ETN, ADA, and JAKi initiators at 6- and 12-months follow-up. Table S4. Unadjusted change in disease activity and PROs among first-line initiators of ETN, ADA, or JAKi at 6- and 12- months of follow-up. Table S5. Unadjusted change in disease activity and PROs among first-line initiators of ETN, ADA, and JAKi monotherapy at 6- and 12-months of follow-up. Table S6. Adjusted change in disease activity and PROs among first-line initiators of ADA and JAKi monotherapy at 6- and 12- months of follow-up relative to ETN. [file 13075_2023_3120_MOESM1_ESM.docx]

**Treatment patterns and clinical outcomes in patients with rheumatoid arthritis initiating etanercept, adalimumab, or Janus kinase inhibitor as first-line therapy: results from the real-world CorEvitas RA Registry**

Dimitrios A. Pappas, Jacqueline O’Brien, Lin Guo, Ying Shan, Joshua F. Baker, Gregory Kricorian, Scott Stryker, David H. Collier

**Additional File 1: Supplementary Tables**

**Table S1. Demographics and clinical characteristics at index visit for first-line ETN, ADA, and JAKi initiators with 12 months of follow-up**

| **Characteristic** | **ETN initiators N = 589** | **ADA initiators N = 749** | **JAKi initiators**  **N = 264** |
| --- | --- | --- | --- |
| Age, years, mean (SD) | 55.2 (12.3) | 56.1 (12.1) | 61.4 (12.6) |
| Women, n (%) | 443 (75.2) | 569 (76.0) | 204 (77.3) |
| White, n/N (%) | 472/585 (80.7) | 620/743 (83.4) | 220/261 (84.3) |
| BMI, kg/m^2^, mean (SD) | 30.9 (7.7) | 31.3 (7.9) | 30.3 (7.2) |
| Duration of RA, years, mean (SD) | 5.9 (7.7) | 5.7 (7.1) | 8.2 (9.5) |
| Rheumatoid factor positive, n/N (%) | 241/355 (67.9) | 322/491 (65.6) | 107/166 (64.5) |
| CCP positive, n/N (%) | 234/355 (65.9) | 317/471 (67.3) | 106/170 (62.4) |
| College education or above, n (%) | 343 (60.4) | 390 (53.8) | 138 (54.8) |
| History of comorbidities, n (%) | | | |
| Cardiovascular disease | 69 (11.7) | 93 (12.4) | 38 (14.4) |
| Malignancy | 23 (3.9) | 34 (4.5) | 29 (11.0) |
| Serious infections | 33 (5.6) | 50 (6.7) | 23 (8.7) |
| Fractures | 144 (24.4) | 244 (32.6) | 84 (31.8) |
| Deep vein thrombosis/ pulmonary embolism | 7 (1.2) | 15 (2.0) | 4 (1.5) |
| Medication history | | | |
| Prior number of csDMARDs received (including current csDMARD), mean (SD) | 1.6 (0.8) | 1.8 (0.9) | 1.7 (1.0) |
| History of prednisone use, n (%) | 320 (54.3) | 401 (53.5) | 134 (50.8) |
| Current therapy | n = 589 | n = 749 | n = 264 |
| Monotherapy, n (%) | 136 (23.1) | 143 (19.1) | 77 (29.2) |
| Combination therapy, n (%) | 453 (76.9) | 606 (80.9) | 187 (70.8) |
| MTX, n (%) | 299 (50.8) | 382 (51.0) | 101 (38.3) |
| Non-MTX nbDMARDs,^a^ n (%) | 89 (15.1) | 123 (16.4) | 59 (22.3) |
| MTX and non-MTX nbDMARDs,^a^ n (%) | 65 (11.0) | 101 (13.5) | 27 (10.2) |
| Prednisone use, n (%) | 161 (27.3) | 216 (28.8) | 64 (24.2) |
| Dose of prednisone, mg, mean (SD) | 7.8 (4.8) | 7.3 (5.8) | 6.4 (3.5) |
| Disease activity and PROs, mean (SD) | | | |
| TJC-28 | 6.9 (7.3) | 6.5 (6.7) | 6.0 (6.6) |
| SJC-28 | 5.4 (5.7) | 4.6 (5.0) | 5.0 (5.1) |
| PhGA^b^ | 35.7 (24.6) | 35.2 (23.3) | 32.6 (22.1) |
| PtGA^b^ | 45.3 (28.0) | 45.5 (27.3) | 39.8 (26.4) |
| CDAI | 20.4 (14.7) | 19.1 (13.0) | 18.2 (13.0) |
| Patient pain^b^ | 48.3 (28.6) | 49.0 (28.7) | 42.0 (28.6) |
| Patient fatigue^b^ | 47.9 (31.1) | 47.3 (29.5) | 44.8 (30.9) |
| mHAQ | 0.5 (0.5) | 0.5 (0.5) | 0.5 (0.5) |
| EQ-5D | 0.7 (0.2) | 0.7 (0.2) | 0.7 (0.2) |
| Patients with morning stiffness, n (%) | 482 (87.6) | 572 (85.6) | 198 (81.8) |
| Duration of morning stiffness, hours,  mean (SD) | 2.2 (3.9) | 2.1 (3.7) | 2.0 (3.8) |

“n” represents the number of patients with available data at the index visit.
^a^ nbDMARDs include methotrexate, hydroxychloroquine, leflunomide, sulfasalazine, azathioprine, minocycline, and cyclosporine.
^b^ Visual analog scale (0–100).

ADA, adalimumab; BMI, body mass index; CCP, cyclic citrullinated peptide; CDAI, Clinical Disease Activity Index; csDMARD, conventional synthetic disease-modifying antirheumatic drug; EQ-5D, EuroQol-5D; ETN, etanercept; JAKi, Janus kinase inhibitor, mHAQ, modified Health Assessment Questionnaire; MTX, methotrexate; PhGA, Physician’s Global Assessment of Disease Activity; PRO, patient-reported outcome; PtGA, Patient’s Global Assessment of Disease Activity; RA, rheumatoid arthritis; SD, standard deviation; SJC-28, swollen joint count of 28 joints; TJC-28, tender joint count of 28 joints.

**Table S2. Demographics and clinical characteristics at index visit for first-line ETN, ADA, and JAKi monotherapy initiators with 12 months of follow-up**

| **Characteristic** | **ETN initiators N = 136** | **ADA initiators N = 143** | **JAKi initiators**  **N = 77** |
| --- | --- | --- | --- |
| Age, years, mean (SD) | 53.7 (13.4) | 55.3 (11.7) | 62.1 (13.6) |
| Women, n (%) | 98 (72.1) | 100 (69.9) | 59 (76.6) |
| White, n/N (%) | 109/136 (80.1) | 120/141 (85.1) | 65/76 (85.5) |
| BMI, kg/m^2^, mean (SD) | 30.7 (7.6) | 30.8 (7.0) | 29.8 (6.5) |
| Duration of RA, years, mean (SD) | 5.8 (7.6) | 5.6 (6.5) | 8.0 (10.2) |
| Rheumatoid factor positive, n/N (%) | 53/75 (70.7) | 54/91 (59.3) | 26/44 (59.1) |
| CCP positive, n/N (%) | 57/79 (72.2) | 56/89 (62.9) | 28/49 (57.1) |
| College education or above, n (%) | 83 (61.9) | 75 (54.7) | 38 (50.7) |
| History of comorbidities, n (%) | | | |
| Cardiovascular disease | 12 (8.8) | 20 (14.0) | 8 (10.4) |
| Malignancy | 3 (2.2) | 6 (4.2) | 11 (14.3) |
| Serious infections | 6 (4.4) | 10 (7.0) | 5 (6.5) |
| Fractures | 38 (27.9) | 48 (33.6) | 24 (31.2) |
| Deep vein thrombosis/ pulmonary embolism | 0 (0.0) | 1 (0.7) | 1 (1.3) |
| Medication history, n (%) | | | |
| Prior number of csDMARDs received (including current csDMARD),  mean (SD) | 1.3 (0.9) | 1.6 (1.1) | 1.3 (1.0) |
| History of prednisone use, n (%) | 66 (48.5) | 71 (49.7) | 38 (49.4) |
| Prednisone use, n (%) | 36 (26.5) | 32 (22.4) | 19 (24.7) |
| Dose of prednisone, mg, mean (SD) | 9.3 (5.9) | 8.5 (5.7) | 7.6 (5.0) |
| Disease activity and PROs, mean (SD) | | | |
| TJC-28 | 5.8 (6.4) | 5.8 (6.5) | 6.1 (7.2) |
| SJC-28 | 4.9 (4.7) | 3.6 (4.5) | 4.8 (4.9) |
| PhGA^a^ | 37.4 (24.3) | 32.8 (24.0) | 29.0 (21.9) |
| PtGA^a^ | 47.7 (27.5) | 46.1 (26.7) | 38.8 (28.5) |
| CDAI | 19.2 (13.1) | 17.2 (12.8) | 17.7 (13.7) |
| Patient pain^a^ | 50.3 (28.5) | 52.6 (29.7) | 38.2 (29.5) |
| Patient fatigue^a^ | 45.1 (32.5) | 50.6 (29.0) | 45.7 (32.4) |
| mHAQ | 0.5 (0.5) | 0.6 (0.5) | 0.5 (0.6) |
| EQ-5D | 0.7 (0.2) | 0.7 (0.2) | 0.8 (0.2) |
| Patients with morning stiffness, n (%) | 113 (89.0) | 109 (83.2) | 54 (80.6) |
| Duration of morning stiffness, hours,  mean (SD) | 2.4 (4.2) | 2.4 (4.2) | 2.1 (4.5) |

“n” represents the number of patients with available data at the index visit.
^a^ Visual analog scale (0–100).

ADA, adalimumab; BMI, body mass index; CCP, cyclic citrullinated peptide; CDAI, Clinical Disease Activity Index; csDMARD, conventional synthetic disease-modifying antirheumatic drug; EQ-5D, EuroQol-5D; ETN, etanercept; JAKi, Janus kinase inhibitor, mHAQ, modified Health Assessment Questionnaire; PhGA, Physician’s Global Assessment of Disease Activity; PtGA, Patient’s Global Assessment of Disease Activity; PRO, patient-reported outcome; RA, rheumatoid arthritis; SD, standard deviation; SJC-28, swollen joint count of 28 joints; TJC-28, tender joint count of 28 joints.

**Table S3. Reasons for discontinuation of treatment among ETN, ADA, and JAKi initiators at 6- and 12-months follow-up**

| **Category of reason for discontinuation, n (%)** | **At 6 months follow-up** | | | **At 12 months follow-up** | | |
| --- | --- | --- | --- | --- | --- | --- |
|  | **ETN initiators N = 183** | **ADA initiators N = 233** | **JAKi initiators**  **N = 80** | **ETN initiators N = 181** | **ADA initiators N = 237** | **JAKi initiators**  **N = 73** |
| Efficacy | 83 (45.4) | 118 (50.6) | 23 (28.7) | 93 (51.4) | 117 (49.4) | 28 (38.4) |
| Safety | 52 (28.4) | 70 (30.0) | 27 (33.8) | 38 (21.0) | 64 (27.0) | 20 (27.4) |
| Cost/insurance | 19 (10.4) | 16 (6.9) | 16 (20.0) | 21 (11.6) | 17 (7.2) | 17 (23.3) |
| Other | 36 (19.7) | 42 (18.0) | 18 (22.5) | 34 (18.8) | 47 (19.8) | 12 (16.4) |

N refers to the number of patients with treatment discontinuation reason reported. Subjects can report up to 3 reasons for discontinuation of treatment. Up to 3 reasons for discontinuation of treatment could be reported; hence, the total may exceed 100%. Efficacy reasons included (but were not limited to) inadequate initial response (primary failure), failure to maintain initial response (secondary failure). Safety reasons included (but were not limited to) major side effects, minor side effects, and fear of future side effects. Cost/insurance reason included lack of insurance. Other reasons included (but were not limited to) frequency or route of administration and patient preference.

**Table S4. Unadjusted change in disease activity and PROs among first-line initiators of ETN, ADA, or JAKi at 6- and 12- months of follow-up**

| **Outcome** | **At 6 months** | | | **At 12 months** | | |
| --- | --- | --- | --- | --- | --- | --- |
|  | **ETN initiators**  **N = 803** | **ADA initiators  N = 984** | **JAKi initiators  N = 361** | **ETN initiators**  **N = 589** | **ADA initiators  N = 749** | **JAKi initiators  N = 264** |
| CDAI | n = 750  6.9 (5.9, 7.8) | n = 901  6.4 (5.6, 7.2) | n = 332  4.7 (3.3, 6.0) | n = 549  7.4 (6.2, 8.5) | n = 672  6.1 (5.1, 7.1) | n = 242  5.1 (3.5, 6.8) |
| TJC-28 | n = 757  2.5 (2.0, 3.0) | n = 905  2.4 (2.0, 2.8) | n = 333  1.8 (1.1, 2.4) | n = 553  2.7 (2.2, 3.3) | n = 675  2.3 (1.7, 2.8) | n = 242  1.9 (1.1, 2.8) |
| SJC-28 | n = 757  2.2 (1.9, 2.6) | n = 905  1.8 (1.5, 2.1) | n = 333  1.6 (1.1, 2.1) | n = 553  2.4 (2.0, 2.8) | n = 675  1.8 (1.5, 2.2) | n = 242  1.6 (1.0, 2.2) |
| PhGA | n = 758  13.6 (11.9, 15.3) | n = 907  13.2 (11.6, 14.7) | n = 338  9.1 (6.7, 11.4) | n = 553  14.6 (12.4, 16.7) | n = 674  12.6 (10.8, 14.4) | n = 242  9.2 (6.2, 12.1) |
| PtGA | n = 757  9.0 (7.0, 11.1) | n = 906  8.7 (6.9, 10.5) | n = 339  5.7 (2.8, 8.5) | n = 551  8.0 (5.7, 10.3) | n = 674  8.5 (6.3, 10.7) | n = 244  6.8 (3.3, 10.2) |
| mHAQ | n = 740  0.12 (0.09, 0.15) | n = 866  0.09 (0.07, 0.12) | n = 330  0.06 (0.02, 0.10) | n = 537  0.10 (0.07, 0.14) | n = 637  0.07 (0.04, 0.10) | n = 235  0.06 (0.01, 0.11) |
| Patient pain | n = 758  9.7 (7.5, 11.9) | n = 906  10.6 (8.8, 12.5) | n = 339  8.9 (5.8, 12.1) | n = 551  8.8 (6.4, 11.3) | n = 674  8.7 (6.5, 11.0) | n = 244  7.5 (3.9, 11.1) |
| Patient fatigue | n = 756  6.7 (4.7, 8.8) | n = 899  4.9 (3.1, 6.6) | n = 337  6.1 (3.2, 8.9) | n = 551  7.4 (4.8, 10.0) | n = 669  5.2 (3.0, 7.3) | n = 243  7.3 (4.0, 10.7) |
| EQ-5D | n = 684  –0.04  (–0.05, –0.03) | n = 830  –0.03  (–0.05, –0.02) | n = 308  –0.03  (–0.05, –0.01) | n = 504  –0.04  (–0.05, –0.02) | n = 626  –0.04  (–0.05, –0.02) | n = 221  –0.04  (–0.06, –0.01) |
| Morning stiffness hours | n = 752  0.77 (0.48, 1.06) | n = 900  0.44 (0.22, 0.67) | n = 331  0.38 (0.04, 0.71) | n = 548  0.51 (0.14, 0.88) | n = 668  0.40 (0.13, 0.67) | n = 240  0.34 (–0.04, 0.71) |
| Achievement of LDA,^a^  n/N (%) | 260/599 (43.4) | 312/744 (41.9) | 88/271 (32.5) | 179/437 (41.0) | 224/565 (39.6) | 74/193 (38.3) |
| Achievement of remission,^b^ n/N (%) | 136/746 (18.2) | 140/926 (15.1) | 39/338 (11.5) | 103/547 (18.8) | 117/700 (16.7) | 43/248 (17.3) |
| Achievement of MCID in CDAI,^c^ n/N (%) | 349/750 (46.5) | 431/901 (47.8) | 126/332 (38.0) | 243/549 (44.3) | 280/672 (41.7) | 107/242 (44.2) |

Values represent the mean (95% CI), unless specified. The mean change from baseline was calculated by subtracting the value at the follow-up visit from the value at the baseline. Positive value indicates improvement from baseline, while negative value indicates worsening from baseline.

^a^ CDAI score ≤ 10 among those with moderate or high disease activity at baseline.

^b^ CDAI score ≤ 2.8 among those with low disease activity or more severe disease activity.

^c^ MCID is defined as a decrease in CDAI score of > 1, > 6, and > 12 for those in LDA (CDAI ≤ 10), MDA (CDAI > 10–22), and had (CDAI > 22) at baseline, respectively.
ADA, adalimumab; CDAI, Clinical Disease Activity Index; CI, confidence interval; EQ-5D, EuroQol-5D; ETN, etanercept; JAKi, Janus kinase inhibitor; LDA, low disease activity; MCID, minimum clinically important difference; mHAQ, modified Health Assessment Questionnaire; OR, odds ratio; PhGA, Physician’s Global Assessment of Disease Activity; PRO, patient-reported outcome; PtGA, Patient’s Global Assessment of Disease Activity; SJC‑28, swollen joint count of 28 joints; TJC-28, tender joint count of 28 joints.**Table S5. Unadjusted change in disease activity and PROs among first-line initiators of ETN, ADA, and JAKi monotherapy at 6- and 12-months of follow-up**

| **Outcome** | **At 6 months** | | | **At 12 months** | | |
| --- | --- | --- | --- | --- | --- | --- |
|  | **ETN initiators  N = 197** | **ADA initiators  N = 191** | **JAKi initiators  N = 117** | **ETN initiators**  **N = 136** | **ADA initiators  N = 143** | **JAKi initiators  N = 77** |
| CDAI | n = 180  6.1 (4.1, 8.2) | n = 175  4.2 (2.6, 5.9) | n = 104  4.8 (1.9, 7.7) | n = 126 5.6 (3.3, 7.9) | n = 131  4.3 (2.2, 6.3) | n = 67  3.8 (0.6, 7.0) |
| TJC-28 | n = 182  2.2 (1.3, 3.2) | n = 176  1.6 (0.8, 2.5) | n = 104  1.9 (0.4, 3.3) | n = 127  1.9 (1.0, 2.9) | n = 131  1.7 (0.7, 2.7) | n = 67  1.2 (–0.6, 2.9) |
| SJC-28 | n = 182  2.1 (1.3, 2.8) | n = 176  0.9 (0.3, 1.5) | n = 104  1.6 (0.6, 2.6) | n = 127  1.7 (0.7, 2.6) | n = 131  1.2 (0.6, 1.9) | n = 67  1.6 (0.3, 2.8) |
| PhGA | n = 181  13.7 (9.7, 17.6) | n = 176  10.4 (6.7, 14.0) | n = 105  8.1 (3.6, 12.7) | n = 127  15.0 (10.4, 19.5) | n = 131  10.0 (6.1, 14.0) | n = 67  5.0 (0.2, 9.9) |
| PtGA | n = 181  6.4 (1.8, 11.0) | n = 176  6.9 (2.8, 11.0) | n = 107  6.9 (2.0, 11.9) | n = 126  7.0 (1.9, 12.1) | n = 132  4.7 (0.4, 9.0) | n = 68  6.3 (0.3, 12.3) |
| mHAQ | n = 175  0.12 (0.05, 0.19) | n = 171  0.07 (0.01, 0.12) | n = 106  0.08 (0.02, 0.15) | n = 121  0.09 (0.00, 0.17) | n = 128 0.05 (–0.01, 0.11) | n = 67  0.06 (–0.02, 0.14) |
| Patient pain | n = 181  7.5 (2.7, 12.2) | n = 176  9.4 (5.0, 13.9) | n = 107  6.4 (0.7, 12.0) | n = 126 8.0 (2.3, 13.7) | n = 132  8.4 (3.5, 13.4) | n = 68  1.5 (–5.3, 8.3) |
| Patient fatigue | n = 179  3.0 (–1.3, 7.3) | n = 175  5.2 (1.1, 9.3) | n = 107  5.1 (0.1, 10.2) | n = 126 2.9 (–2.8, 8.6) | n = 132  4.1 (–0.6, 8.7) | n = 68 4.7 (–0.2, 9.5) |
| EQ-5D | n = 157  –0.03  (–0.06, 0.00) | n = 162  –0.02  (–0.04, 0.01) | n = 98  –0.04  (–0.06, –0.01) | n = 116 –0.04  (–0.07, 0.00) | n = 123 0.00  (–0.03, 0.03) | n = 63 –0.04  (–0.08, 0.00) |
| Morning stiffness hours | n = 179  0.74 (0.05, 1.42) | n = 174  0.12 (–0.52, 0.77) | n = 105  0.07 (–0.73, 0.88) | n = 125  0.46 (–0.38, 1.30) | n = 131  0.52 (–0.31, 1.35) | n = 67  –0.23 (–0.64, 0.19) |
| Achievement of LDA,^a^  n/N (%) | 73/151 (48.3) | 53/135 (39.3) | 30/85 (35.3) | 47/105 (44.8) | 32/100 (32.0) | 19/55 (34.5) |
| Achievement of remission,^b^ n/N (%) | 31/184 (16.8) | 27/176 (15.3) | 14/107 (13.1) | 17/125 (13.6) | 13/127 (10.2) | 14/72 (19.4) |
| Achievement of MCID in CDAI,^c^ n/N (%) | 79/180 (43.9) | 76/175 (43.4) | 38/104 (36.5) | 50/126 (39.7) | 45/131 (34.4) | 27/67 (40.3) |

Values represent the mean (95% CI), unless specified. The mean change from baseline was calculated by subtracting the value at the follow-up visit from the value at the baseline. Positive value indicates improvement from baseline, while negative value indicates worsening from baseline.

^a^ CDAI score ≤ 10 among those with moderate or high disease activity at baseline.

^b^ CDAI score ≤ 2.8 among those with low disease activity or more severe disease activity.

^C^ MCID is defined as a decrease in CDAI score of > 1, > 6, and > 12 for those in LDA (CDAI ≤ 10), MDA (CDAI > 10–22), and HDA (CDAI > 22) at baseline, respectively.

ADA, adalimumab; CDAI, Clinical Disease Activity Index; CI, confidence interval; EQ-5D, EuroQol-5D; ETN, etanercept; JAKi, Janus kinase inhibitor; LDA, low disease activity; MCID, minimum clinically important difference; mHAQ, modified Health Assessment Questionnaire; OR, odds ratio; PhGA, Physician’s Global Assessment of Disease Activity; PRO, patient-reported outcome; PtGA, Patient’s Global Assessment of Disease Activity; SJC‑28, swollen joint count of 28 joints; TJC-28, tender joint count of 28 joints.

**Table S6. Adjusted change in disease activity and PROs among first-line initiators of ADA and JAKi monotherapy at 6- and 12- months of follow-up relative to ETN**

| **Outcome** | **At 6 months^a^** | | **At 12 months^b^** | |
| --- | --- | --- | --- | --- |
|  | **ADA initiators** | **JAK initiators** | **ADA initiators** | **JAK initiators** |
| CDAI | –0.84 (–3.31, 1.62) | 0.67 (–2.22, 3.56) | –0.75 (–3.51, 2.02) | 0.12 (–3.23, 3.47) |
| TJC-28 | –0.49 (–1.67, 0.68) | 0.06 (–1.33, 1.44) | –0.49 (–1.83, 0.84) | –0.98 (–2.61, 0.64) |
| SJC-28 | –0.42 (–1.28, 0.43) | –0.27 (–1.28, 0.74) | 0.10 (–0.91, 1.11) | 0.38 (–0.85, 1.61) |
| PhGA | 0.17 (–4.38, 4.73) | 1.30 (–4.08, 6.67) | –3.72 (–8.85, 1.41) | –3.63 (–9.88, 2.61) |
| PtGA | 0.65 (–4.82, 6.12) | 4.75 (–1.76, 11.27) | 0.02 (–5.92, 5.96) | 6.16 (–1.08. 13.41) |
| mHAQ | –0.03 (–0.11, 0.05) | 0.04 (–0.05, 0.13) | –0.02 (–0.11, 0.08) | 0.03 (–0.08, 0.14) |
| Patient pain | 0.75 (–5.09, 6.60) | 4.32 (–2.52, 11.15) | 0.59 (–5.74, 6.92) | 3.14 (–4.53, 10.81) |
| Patient fatigue | 0.40 (–5.16, 5.95) | 4.48 (–2.06, 11.01) | 1.35 (–4.94, 7.65) | 5.25 (–2.36, 12.85) |
| EQ-5D | 0.01 (–0.03, 0.05) | –0.03 (–0.07, 0.01) | 0.03 (–0.01, 0.07) | –0.04 (–0.09, 0.00) |
| Morning stiffness hours | –0.27 (–1.05, 0.50) | –0.32 (–1.23, 0.60) | 0.26 (–0.60, 1.12) | –0.32 (–1.36, 0.72) |
| Achievement of LDA,^c^  OR (95% CI) | 0.88 (0.49, 1.59) | 1.04 (0.50, 2.16) | 0.68 (0.32, 1.45) | 0.69 (0.27, 1.76) |
| Achievement of remission,^d^  OR (95% CI) | 1.13 (0.54, 2.38) | 1.26 (0.53, 2.97) | 1.47 (0.53, 4.09) | 2.77 (0.91, 8.42) |
| Achievement of MCID in CDAI,^e^  OR (95% CI) | 1.10 (0.69, 1.76) | 0.81 (0.46, 1.43) | 0.82 (0.46, 1.45) | 1.11 (0.57, 2.16) |

Values represent regression coefficients and 95% CIs, unless otherwise specified. A positive value for adjusted change indicates a larger degree of improvement relative to the ETN reference.

^a^ Adjusted by baseline covariates including baseline value of the outcome, age, race (White vs non-White), college education, work status (full-time vs part-time vs other), private insurance, smoking status, Medicare, weight, history of CVD, hypertension, diabetes, and fractures, prednisone use, number of csDMARDs used, and CDAI (or the individual components for changes in specific CDAI measures).

^b^ Adjusted by baseline covariates including baseline value of the outcome, age, race (White vs non-White), college education, work status (full-time vs part-time vs other), private insurance, smoking status, Medicare, weight, history of CVD, hypertension, diabetes, and fractures, prednisone use, number of csDMARDs used, and CDAI (or the individual components for changes in specific CDAI measures).

^c^ CDAI score ≤ 10 among those with moderate or high disease activity at baseline.

^d^ CDAI score ≤ 2.8 among those with low disease activity or more severe disease activity.

^e^ MCID is defined as a decrease in CDAI score of > 1, > 6, and > 12 for those in LDA (CDAI ≤ 10), MDA (CDAI > 10–22), and HDA (CDAI > 22) at baseline, respectively.

ADA, adalimumab; CDAI, Clinical Disease Activity Index; CI, confidence interval; csDMARD, conventional synthetic disease-modifying antirheumatic drug; CVD, cardiovascular disease; EQ-5D, EuroQol-5D; ETN, etanercept; JAKi, Janus kinase inhibitor; LDA, low disease activity; MCID, minimum clinically important difference; mHAQ, modified Health Assessment Questionnaire; OR, odds ratio; PhGA, Physician’s Global Assessment of Disease Activity; PRO, patient-reported outcome; PtGA, Patient’s Global Assessment of Disease Activity; SJC-28, swollen joint count of 28 joints; TJC-28, tender joint count of 28 joints.
